# Magnesium Foliar Supplementation Increases Grain Yield of Soybean and Maize by Improving Photosynthetic Carbon Metabolism and Antioxidant Metabolism

**DOI:** 10.3390/plants10040797

**Published:** 2021-04-19

**Authors:** Vitor Alves Rodrigues, Carlos Alexandre Costa Crusciol, João William Bossolani, Luiz Gustavo Moretti, José Roberto Portugal, Tamara Thaís Mundt, Sirlene Lopes de Oliveira, Ariani Garcia, Juliano Carlos Calonego, Romulo Pisa Lollato

**Affiliations:** 1 Department of Crop Science, College of Agricultural Sciences, São Paulo State University (UNESP), Botucatu, SP 18610-034, Brazil; vitorarodrigues1@gmail.com (V.A.R.); souzamoretti@gmail.com (L.G.M.); jose.portugal@unesp.br (J.R.P.); tamarathaism@gmail.com (T.T.M.); sirlene.lopes@unesp.br (S.L.d.O.); ariani_garcia@hotmail.com (A.G.); juliano.calonego@unesp.br (J.C.C.); 2 Throckmorton Plant Science Center, Department of Agronomy, Kansas State University (KSU), 1712 Claflin Road, Manhattan, KS 66506, USA; lollato@ksu.edu

**Keywords:** photosynthesis, oxidative stress, foliar application, sink-source relationship, carbohydrate partitioning

## Abstract

(1) Background: The aim of this study was to explore whether supplementary magnesium (Mg) foliar fertilization to soybean and maize crops established in a soil without Mg limitation can improve the gas exchange and Rubisco activity, as well as improve antioxidant metabolism, converting higher plant metabolism into grain yield. (2) Methods: Here, we tested foliar Mg supplementation in soybean followed by maize. Nutritional status of plants, photosynthesis, PEPcase and Rubisco activity, sugar concentration on leaves, oxidative stress, antioxidant metabolism, and finally the crops grain yields were determined. (3) Results: Our results demonstrated that foliar Mg supplementation increased the net photosynthetic rate and stomatal conductance, and reduced the sub-stomatal CO_2_ concentration and leaf transpiration by measuring in light-saturated conditions. The improvement in photosynthesis (gas exchange and Rubisco activity) lead to an increase in the concentration of sugar in the leaves before grain filling. In addition, we also confirmed that foliar Mg fertilization can improve anti-oxidant metabolism, thereby reducing the environmental stress that plants face during their crop cycle in tropical field conditions. (4) Conclusions: Our research brings the new glimpse of foliar Mg fertilization as a strategy to increase the metabolism of crops, resulting in increased grain yields. This type of biological strategy could be encouraged for wide utilization in cropping systems.

## 1. Introduction

Magnesium (Mg) is present in many essentials physiological processes and play crucial functions on photosynthesis, photoprotection, and on the carbohydrate partitioning within plants [1]. Besides being responsible for chlorophyll synthesis [1], Mg activates diversity enzymes including glutathione synthase, Ribulose 1,5-Bisphosphate (Rubisco) Carboxylase/Oxygenase, phosphoenolpyruvate carboxylase (PEPcase), RNA polymerase, protein kinases, phosphatases, and ATPases [2,3], all essential enzymes for photosynthesis activity and directly influencing plant growth and development.

Several studies have shown that Mg is critically involved in the phloem loading of sucrose and thus carbohydrate partitioning between source and sink tissues [4]. The phloem loading of sucrose is an active process catalyzed by a proton gradient and an H^+^/sucrose co-transporter, and for its proper functioning, great concentration of ATP-Mg is necessary. The ATP-Mg is responsible for the proper functioning of the H^+^ pump located in the plasma membrane [5]. Up to 90% of cytoplasmic Mg concentration is complexed with ATP in plants in which this nutrient is at adequate levels [6,7], justifying the importance an adequate Mg nutrition in plants. The coordination between production and use of photoassimilates for metabolic activity is key for the maintenance of the photosynthetic process and growth by plants [8]. Essentially, the consumption of photoassimilates by the sink organs activates a positive feedback mechanism that stimulates the production of sugar by the leaves, and consequently, photosynthesis activity [9].

A very recently meta-analyzes [10] highlighted the importance of Mg for crop yields in different production systems under varying soil conditions. The authors exhibited that Mg supply increased by 8.5% the mean value of crop yield under field conditions around the world, attributed to the positive effects of Mg fertilization on the plant physiological activities. Since global demand for maize and soybean is forecast to continuing to increase [11,12,13], the use of stimulating supplementary foliar fertilization (i.e., using low doses of foliar fertilizers in areas where there are no nutrient deficiencies) offers an opportunity for further yield improvements. Stimulating fertilization can improve the photosynthetic activity of plants, the translocation of sugars to sink organs, and increase plant growth and yield [4,14,15]. However, the effects of foliar fertilization can be different between soybean and maize plants, as they have different photosynthetic pathways, C_3_ and C_4_, respectively [16]. There is evidence of greater preference for C_4_ plants for heavy Mg isotopes in chlorophyll-a compared to C_3_ plants [17]. This result was attributed to the greater need for energy and the rate of ATP production to fix carbon in C_4_, reducing the energy barrier during the incorporation of Mg to protoporphyrin IX [17]. In addition, Mg foliar spray can also reduce the abiotic stress of crops by protecting the photosynthetic apparatus and activating the antioxidant defense system [18,19,20].

In this context, Mg foliar fertilization in soybean and maize might greatly influence the translocation of carbohydrates to the grains, intensifying this crop production system. However, very a few studies have evaluated the effect of Mg foliar spray on the photosynthetic parameters, plant nutrition, antioxidant metabolism, Rubisco and phosphoenolpyruvate carboxylase (PEPcase) activities, as well as these crop yields under field conditions. Therefore, our study aimed to verify whether Mg foliar fertilization is a viable management practice to assist soybean and maize plants in activating mechanisms that provide greater photosynthetic activity, mitigate environmental stresses, and increase grain yield during two growing seasons under field conditions.

## 2. Results

### 2.1. Weather Conditions

**Figure 1:**
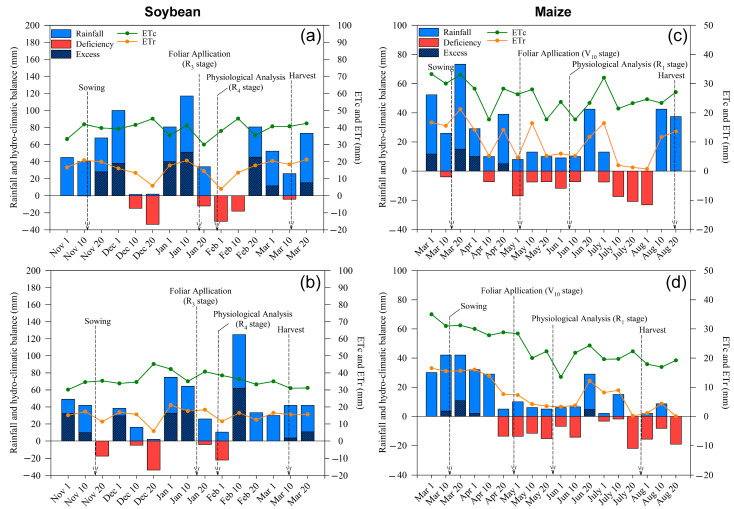
Climatological water balance at Botucatu-SP, Brazil, during the studied soybean ((**a**), 2018/19; (**b**), 2019/20) and maize ((**c**), 2019; (**d**), 2020) crop cycles. ETc, crop evapotranspiration; ETr, real evapotranspiration. The arrows indicate the timing of management operations and sampling.

From soybean sowing to physiological maturity, the total precipitation of the growing season was 569 and 434 mm for the first and second growing seasons. Although the total precipitation was higher in the first growing season, the distribution was less uniform than in the second one. Thus, two periods with low precipitation and consequent dry spell occurred during the first growing season (Figure 1a,b). The first dry spell occurred at the end of the vegetative period (between V_5_ and V_8_ phenological stage) and the beginning of flowering (R_1_ phenological stage), while the second occurred at the end of full flowering (R_2_), lasting until the end of pod formation (R_4_). In the second soybean-growing season, the most striking dry spell occurred during the vegetative period, with a less intense second dry spell occurring between the end of R_2_ and the end of R_4_. These dry periods were of less intensity than those experienced during the first growing season.

During both maize growing seasons, the hydro-climatic balance indicated water scarcity during the majority of the crop’s cycle, with the exception of the first stages of vegetative development (Figure 1c,d). Low precipitation volumes characterized both maize cycles, but drought was more severe during the second growing season (i.e., 319 and 293 mm total precipitation during the first and second growing season, respectively).

### 2.2. Crop Nutrition, Photosynthetic Parameters, and Carbon Assimilation

**Figure 2:**
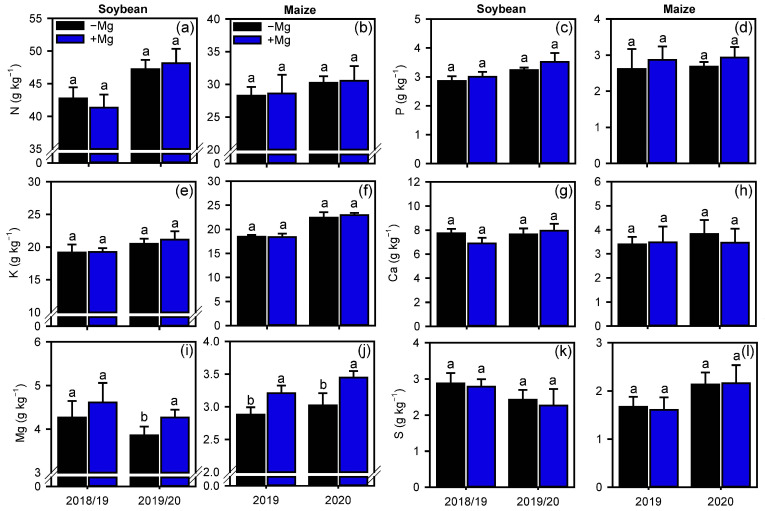
Nutritional status of soybean and maize plants, as indicated by concentrations of N (**a**,**b**), P (**c**,**d**), K (**e**,**f**), Ca (**g**,**h**), Mg (**i**,**j**), and S (**k**,**l**), as affected by foliar Mg fertilization. Different lower-case letters indicate significant differences between treatments (presence or absence of Mg supplementation) by Fisher’s protected least significant difference (LSD) test at *p* ≤ 0.05. Growing seasons was considered as random effects. Error bars express the standard error of the mean (*n* = 4).

Foliar Mg fertilization increased the leaf Mg concentration of soybeans in the 1st growing season (GS), 10.4%, and maize in both growing seasons (1st GS: 13.3%, 2nd GS: 14.2%) (Figure 2i,j). The concentration of other nutrients in the leaves did not change due to the foliar Mg fertilization.

**Figure 3:**
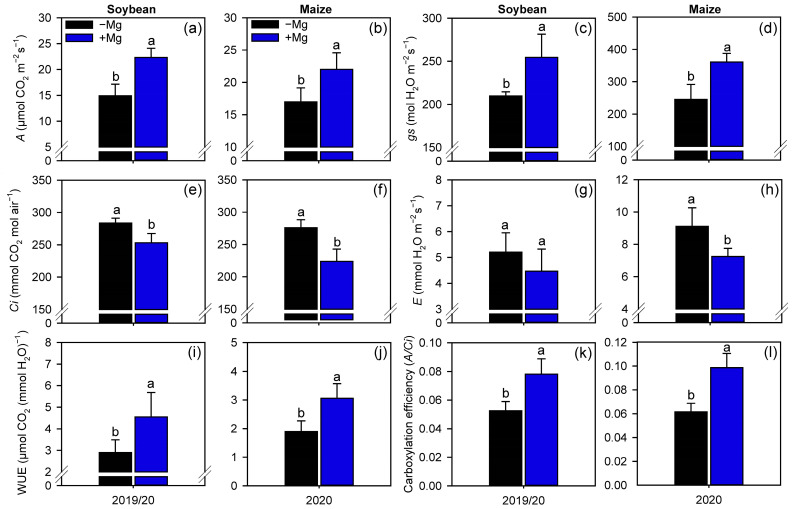
Net photosynthetic rate (**a**,**b**), stomatal conductance (**c**,**d**), substomatal CO_2_ concentration (**e**,**f**), leaf transpiration (**g**,**h**), water use efficiency (**i**,**j**), and carboxylation efficiency (**k**,**l**) of soybean and maize plants as affected by foliar Mg fertilization. Different lower-case letters indicate significant differences between treatments (presence or absence of Mg supplementation) by Fisher’s protected LSD test at *p* ≤ 0.05. Growing seasons was considered as random effects. Error bars express the standard error of the mean (*n* = 4).

Foliar fertilization with Mg improved soybean and maize gas exchange performance, compared with the control treatment where no Mg was applied (Figure 3). Foliar Mg fertilization increased the net photosynthetic rate (*A*), and stomatal conductance (*gs*) by 49% and 21% for soybean, and by 29% and 47% for maize, respectively (Figure 3a,b). Plants treated with Mg also reduced the substomatal CO_2_ concentration (*Ci*) (soybean: 11%, maize: 19%), and leaf transpiration (*E*) of maize by 20% (Figure 3c,d). As a result of increasing *A* and reducing *Ci* and *E*, water use efficiency (WUE) and carboxylation efficiency (*A/Ci*) also increased for both crops (57% and 49% for soybean, and by 62% and 60% for maize, respectively) (Figure 3e,f).

**Figure 4:**
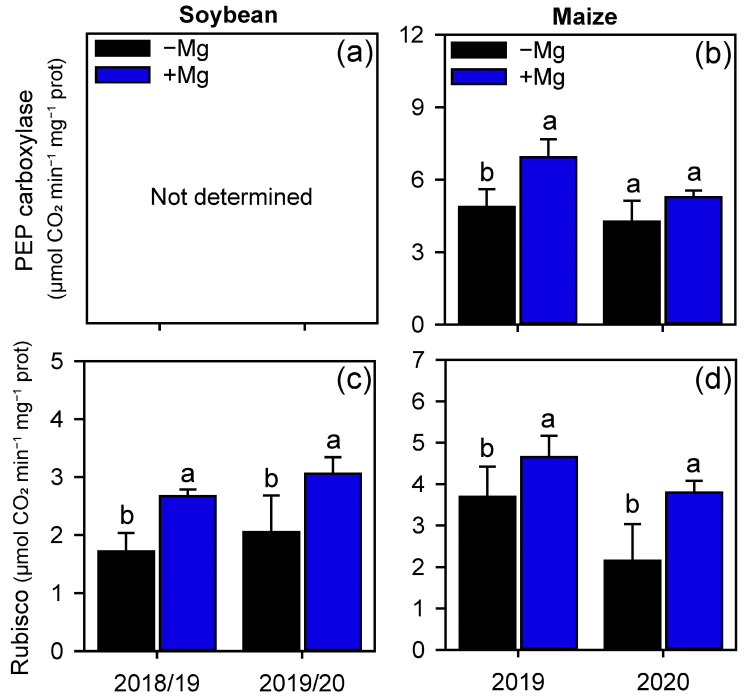
PEP carboxylase (**a**,**b**) and Rubisco (**c**,**d**) activity of soybean and maize plants as affected by foliar Mg fertilization. Different lower-case letters indicate significant differences between treatments (presence or absence of Mg supplementation) by Fisher’s protected LSD test at *p* ≤ 0.05. Growing seasons was considered as random effects. Error bars express the standard error of the mean (*n* = 4).

The enzymes involved in the capture and subsequent fixation of CO_2_ were also increased by Mg foliar fertilization (Figure 4). Phosphoenolpyruvate carboxylase (PEPcase) activity increased in maize only in the 1st GS (42.5%, Figure 4b), but Rubisco activity increased by 55% and 48% on soybean (1st and 2nd GS, respectively), and by 26% and 76% on maize.

**Figure 5:**
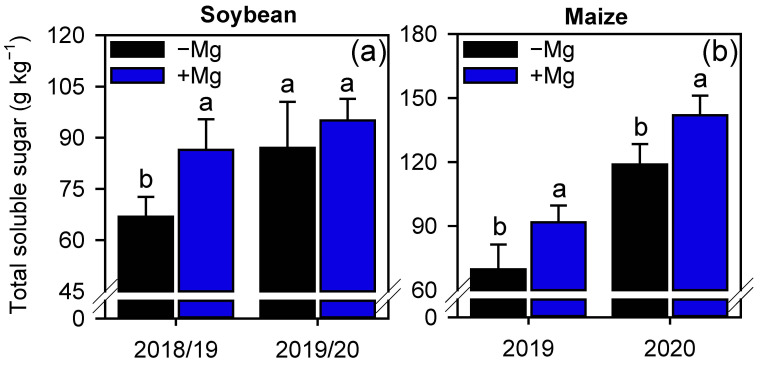
Total soluble sugar concentration in leaves of soybean (**a**) and maize (**b**) plants as affected by foliar Mg fertilization. Different lower-case letters indicate significant differences between treatments (presence or absence of Mg supplementation) by Fisher’s protected LSD test at *p* ≤ 0.05. Growing seasons was considered as random effects. Error bars express the standard error of the mean (*n* = 4).

The increase in photosynthetic parameters mentioned above also increased the concentration of total soluble sugar on leaves of soybean and maize before the grain filling (Figure 5). For soybeans, Mg-treated plants increased the total sugar concentration by 29% compared with the control on the 1st GS (Figure 5a), and for maize, Mg spraying increased total sugar concentration by 31% and 20%, respectively, for 1st and 2nd GS (Figure 5b).

### 2.3. Oxidative Stress and Antioxidant Enzymes

**Figure 6:**
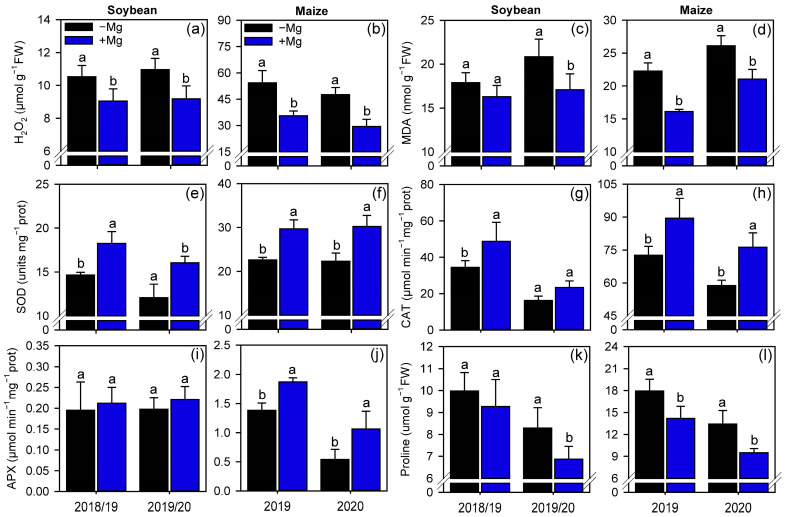
Oxygen peroxide (**a**,**b**), malondialdehyde (**c**,**d**), superoxide dismutase (**e**,**f**), catalase (**g**,**h**), ascorbate peroxidase (**i**,**j**), and proline concentration (**k**,**l**) of soybean and maize plants as affected by foliar Mg fertilization. Different lower-case letters indicate significant differences between treatments (presence or absence of Mg supplementation) by Fisher’s protected LSD test at *p* ≤ 0.05. Growing seasons was considered as random effects. Error bars express the standard error of the mean (*n* = 4).

Oxygen peroxide (H2O2) reduced in soybean (1st GS: 14%, 2nd GS: 16%) and maize (1st GS: 35%, 2nd GS: 38%) plants treated with foliar Mg as compared with the control (Figure 6a,b). The oxidative damage (i.e., lipids peroxidation) in the membrane cells were also reduced when Mg was applied to the leaves of soybean (2nd GS: 18%) and maize (1st GS: 28%, 2nd GS: 19%) (Figure 6c,d). We also measured an increase in the activity of the antioxidant enzymes SOD, CAT, and APX, in both crops. For soybean, foliar spraying with Mg increased the activities of SOD (1st GS: 24.6%, 2nd GS: 33%) and CAT (2nd GS: 42%), but not for APX (Figure 6e,g,i). On the other hand, for maize, Mg-treated plants increased the activities of SOD (1st GS: 31%, 2nd GS: 36%), CAT (1st GS: 23%, 2nd GS: 30%), and APX (1st GS: 35%, 2nd GS: 97%) enzymes (Figure 6f,h,j). Proline concentration in leaves followed the same pattern as that for H2O2 and MDA, reducing in Mg-treated plants for both soybean and maize. For soybean, proline reduction occurred only for the 2nd GS by 17% (Figure 6k), whereas for maize, Mg spraying reduced proline in 21% and 29% for 1st and 2nd GS, respectively (Figure 6l).

### 2.4. Grain Yield

**Figure 7:**
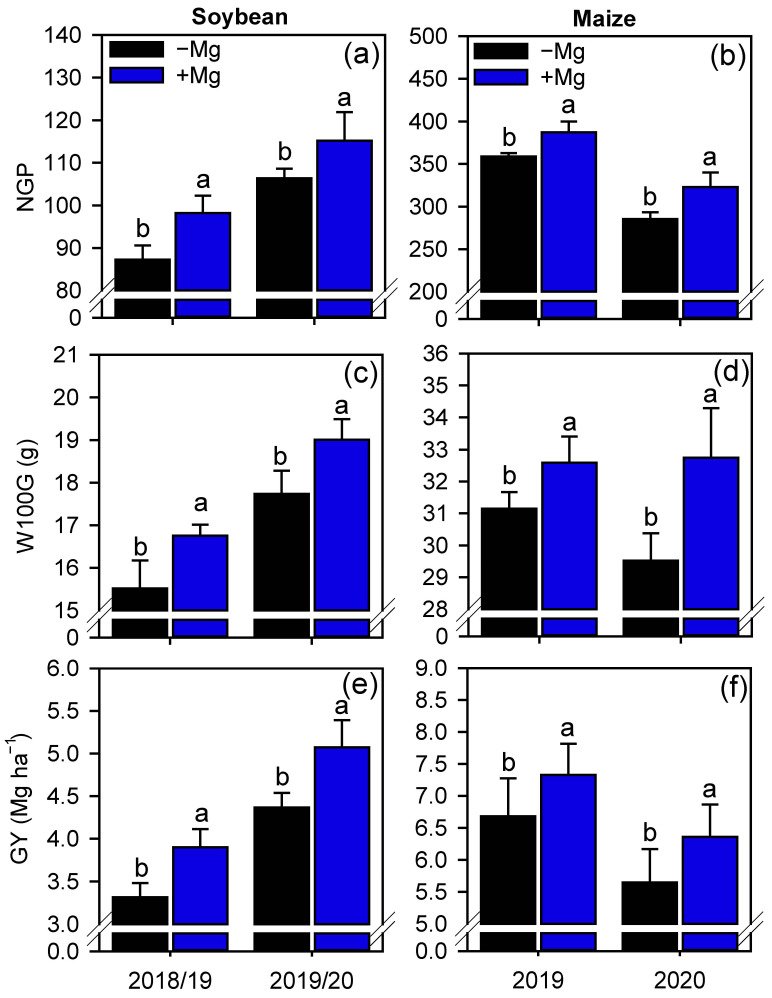
Number of grains per plant (NGP; **a**,**b**), 100-weight grains (W100G; **c**,**d**), and grain yield (GY; **e**,**f**) of soybean and maize as affected by foliar Mg fertilization. Different lower-case letters indicate significant differences between treatments (presence or absence of Mg supplementation) by Fisher’s protected LSD test at *p* ≤ 0.05. Growing seasons was considered as random effects. Error bars express the standard error of the mean (*n* = 4).

Foliar Mg fertilization significantly increased the number of pods per plant of soybean (1st GS: 8.7%, 2nd GS: 9%) (Figure S1a), and consequently, increased the number grains per plant (1st GS: 12.5%, 2nd GS: 8.4%) (Figure 7a), but did not change the number of grains per pod (Figure S1b). For maize, foliar Mg did not change the prolificacy (i.e., ears per plant), but increased the number of grains per ear (1st GS: 7%, 2nd GS: 9%), and as a result, also increased the number of grains per plant (1st GS: 8%, 2nd GS: 13.2%) (Figure 7b). For both crops, the 100-grain weight increased with foliar Mg fertilization (by 8% and 7.2% in the 1st and 2nd GS of soybean, and by 5% and 11% in the two GS of maize) (Figure 7c,d). The positive effects of foliar Mg fertilization on yield components in both crops resulted in increased grain yield by 17% and 16% for 1st and 2nd GS of soybeans, and 9.7% and 12% for the two GS of maize.

### 2.5. Pearson’s Correlation and PCA among Soybean and Maize Parameters

**Figure 8:**
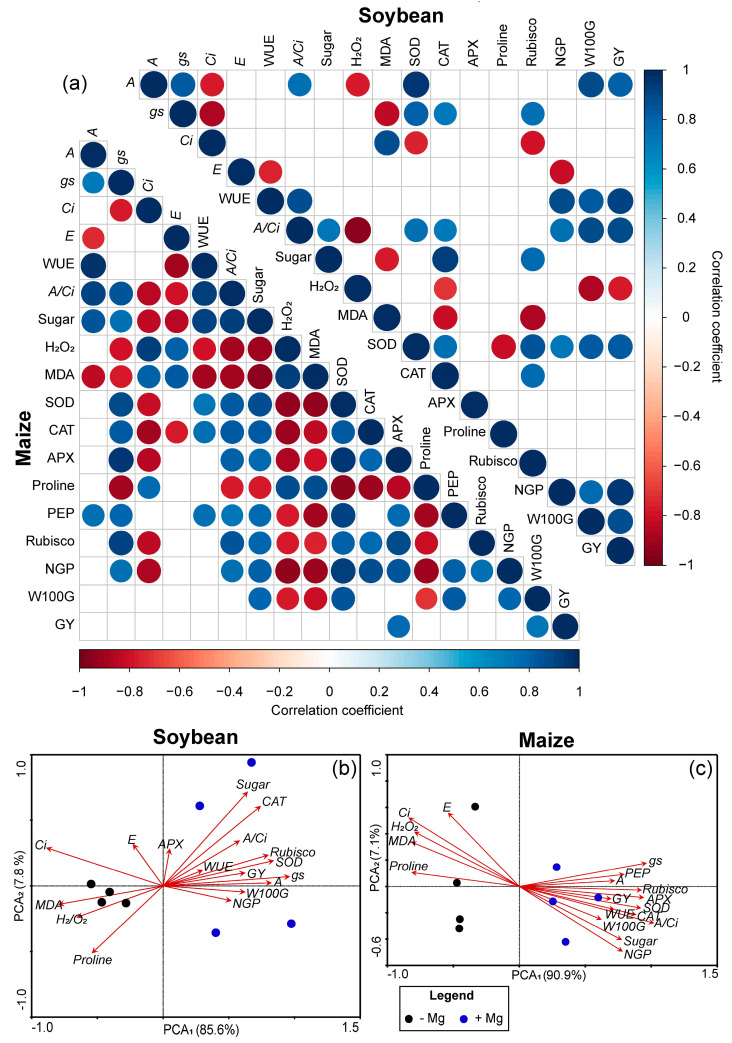
Heatmap of Pearson’s Correlation coefficients and Principal Component Analysis (PCA) among physiological and reproductive parameters of soybean and maize plants. In the Heatmap, only significant correlations at *p* ≤ 0.05 are shown. Net photosynthesis rate (*A*), stomatal conductance (*gs*), internal CO_2_ concentration (*Ci*), leaf transpiration (*E*) water use efficiency (WUE), carboxylation efficiency (*A*/*Ci*), leaf total sugar concentration (Sugar), hydrogen peroxide (H_2_O_2_), malondialdehyde (MDA), superoxide dismutase (SOD), catalase (CAT), ascorbate peroxidase (APX), PEP carboxylase (PEP), number of grains per plant (NGP), weight of 100 grains (W100G), and grain yield (GY).

A greater number of correlations between the evaluated parameters occurred for maize as compared to soybeans (Figure 8a). For soybeans, strong positive correlations occurred among A, gs, A/Ci, SOD, W100G, and GY, as well as negative correlations from these parameters and H_2_O_2_ and MDA. For maize, the impact of the variables related to photosynthesis were greater among themselves and against those related to antioxidant metabolism, when compared to the soybean results.

A clear segregation occurred between the Mg treated and untreated plants for both soybean and maize on PCA (Figure 8b,c). The effect of treatments on the variables demonstrated that the impact was similar in both crops, although in maize, the vectors showed greater uniformity in the spatial distribution of the variables than in soybeans, corroborating the greater number of correlations that occurred in the Pearson’s correlation of maize (Figure 8a).

## 3. Discussion

Magnesium (Mg) is a macronutrient that plays several important roles in plant metabolism [21,22,23]. Interestingly, the vast majority of studies involving foliar Mg fertilization were carried out under controlled conditions, aiming to demonstrate how leaf Mg supplementation can mitigate environmental stresses [24,25]. However, there are a few studies aimed at demonstrating how the additional application of Mg to the leaves can improve photosynthesis, the antioxidative response, and increase the crops yield even when the soil has Mg levels considered adequate for plant development under field conditions.

Although foliar Mg fertilization did not alter the concentration of most leaf macronutrients, it increased the Mg concentration of soybean and maize leaves, especially in the 2nd GS. This change is related to the additional application of Mg combined with to the rapid absorption of the element by the leaves and its high mobility in the phloem [26]. This increased Mg concentration may improve growth parameters due to the important roles that Mg plays in plant metabolism, especially those related to chlorophyll biosynthesis, photosynthesis, and carbon assimilation, in addition to activating numerous key chloroplast enzymes [21,23,27]. Interestingly, in most cases, it is difficult to change the leaf concentration of macronutrients through foliar spraying [28]; however, even with the absence of effect on plant nutrition, additional supply of macroelements during periods of high need by plants, may have increased the plant metabolism [29].

As a result of the additional Mg supply, all the gas exchange parameters, which are closely linked to the activity of PEPcase (on maize) and, especially, Rubisco (in both crops), increased, culminating also in greater leaf concentration of sugars. The beginning of this chain effect may be related to the fact that Mg is a constituent of the chlorophyll, a substance responsible for harvesting light energy [30,31]. Although chlorophyll concentration has not been determined, numerous studies have reported the role of Mg (applied via soil or to the leaves) in increasing the levels of chlorophyll in plants [30,32,33]. During the photosynthetic process, CO_2_ is used as the substrate for photosynthetic assimilation [34,35]. The CO_2_ diffuses into the plant cell through the stomata, therefore, plants with higher stomatal conductance have greater ability to balance the CO_2_ uptake with water loss through transpiration [19]. Our results were based on one-time-point after the Mg supplementation; however, the persistent effects of Mg supplying on gas exchange and photosynthetic enzymes during crop cycle should be considered in future studies. Understanding the persistence of the Mg effects can help in programs for managing Mg-reapplications during the crop cycle.

Our results demonstrated that plants foliar treated with Mg have higher *gs* and lower *E*, resulting in higher *A*, as well as greater WUE. In line with these results, our work also showed that Mg-treated plants had lower *Ci* and, consequently, higher carboxylation efficiency (*A*/*Ci*). These results are derived from the greater activity of PEPcase in maize and, mainly, Rubisco in both crops. The PEPcase activity occurs especially in plants with C_4_ and CAM metabolism [36,37]. In these plants, atmospheric CO_2_ penetrates from the stoma to the mesophyll cells, where it is fixed in an organic acid with 3 C, the phosphoenolpyruvate (PEP), which is transformed into organic acids of 4 C by PEPcase [35,38]. Subsequently, the 4 C organic acid is decarboxylated in the perivascular sheath, where Rubisco fixes the released CO_2_ [35,37] avoiding photorespiration [39].

On the other hand, Rubisco is present in every organism capable of performing CO_2_ photosynthesis [38,40] being responsible for transforming the CO_2_ fixed in sugars. The supplied Mg was applied directly to the leaves, and is readily available for use. Numerous studies reported the positive effect of Mg supply on net photosynthetic rate of various species and under various growing conditions [22,41,42,43,44]. Magnesium directly affects the Rubisco activation [45] by binding to the carbamylated Rubisco chain and to the catalytic chaperone Rubisco activase [46].

In summary, the Mg-treated plants were more efficient photosynthetically, transforming CO_2_ into carbon skeletons (sugar) and losing less water during the process. As a result, both soybean and maize crops converted the sugar produced by photosynthesis into a higher number of grains per plant (due to the high number of pods per plants for soybean and high number of grains per ear of maize), and also in higher 100-grain weight. Interestingly, Mg acts in several processes that modulate the production and translocation of carbohydrates in plants [47,48]. The long-distance transport of carbohydrates (sugars) from source-to-sink organs is carried out via phloem, and this is strongly affected by the availability of Mg [49,50]. This fact indicates that the highest content of total sugars present in the soybean and maize leaves evaluated prior to grain filling, was efficiently carried to the sink organs, culminating in a higher number of grains and with higher weight. The combination of these two parameters resulted in higher grain yield.

Spraying Mg to the crops not only is important for improving photosynthesis and plant growth but also contributes to the improvement of antioxidant metabolism. The cropping of soybean occurs during the spring/summer seasons, which in tropical regions corresponds to the months of September–March. While this period is usually hot and rainy in tropical regions, short dry spells occur frequently during soybean cropping [51], causing a short period of stress due to lack of water and or high temperatures [52], especially in the phenological stages after flowering, as observed in our study (Figure 1). In the case of maize, its cropping occurs during the autumn/winter seasons (April-August), a period characterized by mild temperatures and a dry climate. These meteorological characteristics limit the productivity of maize in the off-season, especially due to long periods with low water availability [53], which also occurred during our study (Figure 1).

Numerous studies reported the beneficial effect of Mg spraying to relieve environmental stresses in crops, such as drought stress [29], heat stress [20,27], and soil acidity stress [54], and temperature [30] by improving the antioxidant metabolism and reducing the cell damages. During the drought stress, there is a reduction in the use of the energy captured in the photosynthesis reaction centers [55]. In plants with limited Mg concentration, the low CO_2_ assimilation reduces the energy consumption (ATP) and reducing power (NADPH) obtained and stored during photosynthesis, limiting utilization of light energy [23]. In this case, the excess energy accumulated in the photosystem results in the overreduction in the electron transport chain [2], leading to a production of reactive oxygen species (ROS) such as H_2_O_2_, and increasing the MDA production, a by-product of cell damages [56,57,58] as occurred in our study. In addition, proline content also increased in untreated plants, reinforcing that there was an increase in stress in these plants [59]. The proline compound is an important nitrogen source produced and used by plants to recover from abiotic stresses and restore their growth [60]. Magnesium applied directly to the leaves increases photosynthetic efficiency, consuming excess energy in the photosystem and reducing the production of ROS [25]. Our results demonstrate that even in the absence of limited Mg levels in the soil and, consequently, adequate Mg levels in the soybean and maize leaves, under environmental stresses, the additional application of Mg via foliar spraying can be a viable technique to help the plant to alleviate these deleterious effects.

The mitigation of the negative effects of excess ROS that can naturally occur in plants and that can be aggravated during the period of environmental stresses was due to the anticipated increase in the specific SOD activity in Mg-treated plants, suggesting an increased requirement for SOD scavenging in chloroplasts and other cell compartments [61,62]. Similarly, the CAT activity also increased, indicating that after the dismutation of O_2_^−^ into H_2_O_2_ by SOD, it was necessary to increase CAT activity to convert H_2_O_2_ into H_2_O [58,63] in both crops. Interestingly, the increased APX activity occurred only in the maize, perhaps due to climatic conditions being more unfavorable during its cropping, resulting in higher rates of ROS production and requiring the action of a greater range of antioxidant enzymes [57,63], as supported by our correlation analysis and PCA. Our results suggested therefore, that SOD, CAT and APX enzymes played a central protective role in the ROS scavenging in soybean and maize plants treated with foliar supplementation with Mg.

## 4. Materials and Methods

### 4.1. Field Description

Field experiments were conducted under rainfed conditions during the 2018–2019 and 2019–2020 spring/summer growing seasons with soybean and during the 2019 and 2020 autumn/winter off-seasons with maize, at the Lageado Experimental Farm of São Paulo State University (UNESP), in the southeastern region of São Paulo State, Brazil (48°26′ W, 22°51′ S, elevation of 786 m altitude). The experimental area has been under no-tillage system for 12 years. The soil is classified as a Ferralsol [64], which corresponds to the classification as clayey textural class, kaolinitic, thermic Typic Haplorthox [65]. According to the Köppen-Geiger climatic classification system [66], the region has a mesothermic climate (Cwa), that is, a humid subtropical climate with dry winters and hot summers. The average rainfall is 1360 mm year^−1^, and the mean annual air temperature is 20.7 °C (50-year average) [67].

Prior to the establishment of the experiment, the soil water-holding capacity was determined according to the tension table and the Richards extractant chamber methods [68] which allowed for the determination of the soil water potential (ψ_w_). The reference evapotranspiration (ET_0_) was calculated by the Penman–Monteith method [69]. For the calculation of crop evapotranspiration (ETc), the crop coefficient (Kc) for each stage of crop development was used [69]. With this information and the rainfall, and the minimum and maximum air temperature of the experimental area, the climatological water balances were calculated using electronic spreadsheets [70]. Following the Thornthwaite and Mather [71] procedure to obtain the real evapotranspiration (ETr), and the deficiency (soil water deficit) or excess (soil water surplus) were established. The climatological water balance of the two experimental growing seasons is shown in Figure 1.

Soil texture [72] and chemical [73] properties at a depth of 0.00–0.20 m were determined prior to the establishment of the experiment and are presented in Table S1. Lime was applied to increase the base saturation (BS) of the topsoil (0.00–0.20-m depth) to 70% approximately 60 days prior to the beginning of the experiment using dolomitic lime (CaMg(CO_3_)_2_) (280 g kg^−1^ of calcium oxide—CaO, 200 g kg^−1^ of magnesium oxide-MgO, and 81% of calcium carbonate equivalents—%E_CaCO3_) [74].

### 4.2. Experimental Design and Treatment Description

A randomized complete block (RCB) design was used with four replicates. The Mg-Foliar fertilization factor was represented by the presence (+Mg) or absence (−Mg) of Mg application.

The Mg-Foliar fertilization was performed at the R_4_ soybean phenological stage [75] by applying 500 g of magnesium (MgCl_2_; Mag-8^®^; Ubyfol; Uberaba, Brazil) ha^−1^ and a vegetable oil adjuvant (30 mL ha^−1^; Disperse^®^; Ubyfol; Uberaba, Brazil) were diluted in 150 L H_2_O ha^−1^. For maize, the Mg-Foliar fertilization was performed at the V_10_ phenological stage by applying the same dose and vegetable oil adjuvant were diluted in 180 L H_2_O ha^−1^.

Foliar fertilization was carried out using an aerograph atomizer propelled by CO_2_, with working pressure of 1.8 BAR. The boom was composed of six 0.5-m spaced nozzles with flat fan nozzles (TTl11004VP), operated at a height of 0.5 m from the ground and speed of 1 ms^−1^ to mimic the action of a commercial spraying apparatus. Foliar spraying was carried out according to the technical recommendations of the manufacturers.

### 4.3. Field Management

#### 4.3.1. Soybean Crop

Prior the soybean sowing (cultivar TMG 7062 RR; 290,000 plants ha^−1^; Tropical Breeding & Genetics^®^; Cambé, Brazil), the seeds were first treated with fungicides (carboxin + thiram at 100 g + 100 g a.i. 100 kg^−1^ seeds) and later inoculated [76]. Each plot consisted of 10 rows that were 10-m long and spaced 0.45 m apart, covering an area of 45 m^2^. Base fertilization included 300 kg ha^−1^ of 00–20–20 (60 kg ha^−1^ of P_2_O_5_ and 60 kg ha^−1^ of K_2_O) in both growing seasons (1st GS and 2nd GS). The management of weeds, insects, and diseases were carried according to the recommendations [77] when necessary, so these were not limiting factors.

#### 4.3.2. Maize Crop

Each maize (hybrid P3707VYH; 60,000 plants ha^−1^; DuPont Pioneer^®^, Johnston, IA, USA) plot consisted of 10 rows that were 10-m long and spaced 0.45 m apart, covered an area of 45 m^2^. The base fertilization was performed with 300 kg ha^−1^ of 08–28–16 (24 kg ha^−1^ of N, 84 kg ha^−1^ of P_2_O_5_, and 48 kg ha^−1^ of K_2_O) in both off-seasons. At the V_6_ maize phenological stage [78], N–K fertilizers were broadcast over the soil surface at a rate of 100 kg N ha^−1^ as ammonium sulfate, and 20 kg K_2_O ha^−1^ as potassium chloride. Phytosanitary treatments were carried out according to the needs of the maize crop.

### 4.4. Plant Sampling and Laboratory Analyzes

#### 4.4.1. Crop Nutrition

Plant nutritional status was evaluated at the R_4_ phenological stage [75] (full pod) in soybean leaves (in the third fully developed leaf and its petiole from 30 plants in each plot) [79], and at the R_1_ phenological stage [78] (silking) in maize leaves (in the middle third of ear leaf from 10 plants per plot) [80]. Briefly, the plant material was used to determine the nitrogen (N), phosphorus (P), potassium (K), calcium (Ca), Mg, and sulfur (S) concentrations according to the methodology described by Malavolta et al. [81].

#### 4.4.2. Gas Exchange Parameters

Gas exchange was evaluated via nondestructive analysis with a Portable Infrared Gas Analyzer CIRAS-3 Portable Photosynthesis System (PP Systems Inc., Amesbury, MA, USA). In soybean, samples were taken at R_4_ phenological stage from the central leaflet of the third fully expanded leaves and intact trifoliate leaf from the plant apex of the main stem of 10 plants per plot. For maize, samples were taken at R_1_ phenological stage [78] by collecting the middle third of 10 ear leaf each plot. The parameters of the instrument were as follows: 380–400 mol mol^−1^ atmospheric CO_2_, 1100 μmol quanta m^−2^ s^−1^ of photosynthetically active radiation (PAR) supplied by LED lamps, 25–27 °C leaf chamber temperature, and 60–70% relative humidity. The minimum equilibration time for each set of measurements was 3 min.

The measurements were performed between 10:00 a.m. and 12:00 p.m. The following parameters were determined: net photosynthesis rate (*A*; μmol CO_2_ m^−2^ s^−1^), stomatal conductance (*g_S_*; mol H_2_O m^−2^ s^−1^), internal CO_2_ concentration in the substomatal cavity (*Ci*; μmol mol^−1^), transpiration (*E*; mmol H_2_O m^−2^ s^−1^), and instantaneous water use efficiency (WUE; μmol CO_2_ (mmol H_2_O)^−1^) was calculated by the *A*/*E* ratio, and the carboxylation efficiency was calculated by the *A*/*Ci* ratio.

#### 4.4.3. Photosynthetic Enzymes

For photosynthetic enzymes, samples were taken in the same leaflets collected for gas exchange parameters. The phosphoenolpyruvate carboxylase (PEPcase) (EC 4.1.1.31) activity was measured using an enzymatic method coupled to NADH oxidation monitored by spectrophotometer at 340 nm [82]. The enzyme activity was measured by recording the decreased absorbance at 340 nm over 300 s and expressed in μmol min^−1^ mg protein^−1^.

The Ribulose-1,5-biphosphate carboxylase:oxygenase (Rubisco) activity was determined using the same extract used to PEPcase activity, and it was spectrophotometrically measured by rate of NADH oxidation at 340 nm [83]. Rubisco activity was calculated from the difference in the absorbance readings at 0 and 1 min (without removing the cuvette from the spectrophotometer) and expressed in μmol min^−1^ mg protein^−1^.

#### 4.4.4. Total Soluble Sugar Concentration

The total soluble sugar concentrations were determined [84]. The concentrations were based on the standard sucrose curve, and the results are expressed in g kg^−1^.

#### 4.4.5. Oxidative Stress and Antioxidant Enzymes

The same leaflets used to assess the gas exchange parameters were sampled to evaluate the contents of hydrogen peroxide (H_2_O_2_) and malondialdehyde (MDA) as well as the activities of superoxide dismutase (SOD; EC:1.15.1.1), catalase (CAT; EC:1.11.1.6), ascorbate peroxidase (APX; EC:1.11.1.11), and proline content for both crops.

Lipid peroxidation was evaluated [85], and the results were expressed in nanomoles of MDA per gram of fresh weight (FW). The H_2_O_2_ content was determined [86], and the content was calculated based on a calibration curve and expressed in μmol g^−1^ FW. SOD activity was measured [87], and the results were expressed in units mg^−1^ of protein. CAT activity was evaluated [88], and the results were expressed in μmol min^−1^ mg^−1^ of protein. APX activity was measured [89], and the results were expressed in μmol min^−1^ mg^−1^ of protein. Proline content was determined [90], and the results were expressed in μmol g^−1^ of FW [91].

#### 4.4.6. Agronomic Parameters and Grain Yield

At soybean and maize physiological maturity, plants were harvested from a 15-m^2^ area from the central part of each plot. We estimated the number of grains per plant, grain yield (Mg ha^−1^), and 100-weight grain (W100G) (the latter two parameters reported at 13% moisture content). The moisture was determined with an automatic measuring device (Gehaka G650i, Brazil).

### 4.5. Data Analyses

All data were initially analyzed via the Shapiro–Wilk test [92] for normality and the Levene’s test for homoscedasticity [93], both at *p* < 0.05. The data were also tested for sphericity by the Bartlett test [94]. The results indicated that all data were distributed normally (W ≥ 0.95) and exhibited no sphericity. Foliar fertilization factors were considered fixed effects, and growing season, replication, and replication nested within year were considered random effects. Subsequently, the means were subjected to analysis of individual variance (ANOVA) by the F test (*p* ≤ 0.05) and, when significant, analyzed using the Fisher’s protected least significant difference (LSD) at *p* ≤ 0.05. We built a heatmap of the Pearson correlation coefficients (*p* ≤ 0.05) among the measured variables and only the significant correlations are shown. Principal component analysis (PCA) was performed through statistical software Canoco v. 4.5.

## 5. Conclusions

Our study confirmed under field conditions that soybean and maize crops react to foliar Mg fertilization by increasing the net CO_2_ assimilation by PEP carboxylase in maize and by Rubisco in both soybean and maize, increasing the total sugar concentration in source tissues and converting into higher grain yield. In addition, in field conditions, where environmental stresses naturally occur, the application of leaf Mg also reduced the oxidative stress by improving the use of energy accumulated in photosynthesis and by increasing the antioxidant enzymes. In conclusion, the additional Mg foliar spraying in crops established in soils without nutritional limitations should be seen as the new approach for obtaining more metabolically active plants and, consequently, with higher grain yield potential.

## Data Availability

The datasets analyzed during the current study are available from the corresponding author upon reasonable request.

## Supplementary Materials

The following are available online at https://www.mdpi.com/article/10.3390/plants10040797/s1. Figure S1: Number of pods per plant (a) and grains per pod (b) of soybean and prolificacy (c) and number of grains per ear (d) of maize plants as affected by presence or absence of the foliar Mg application. Table S1: Physicochemical and biological attributes (0.0–0.2-m depth) before sowing.
