# Supplementary material for: Magnesium Foliar Supplementation Increases Grain Yield of Soybean and Maize by Improving Photosynthetic Carbon Metabolism and Antioxidant Metabolism
Source: Plants (Basel). 2021 Apr 19;10(4):797. doi: 10.3390/plants10040797 (PMC8072903; doi:10.3390/plants10040797)
Supplement: Supplementary file 1 [file plants-10-00797-s001.zip › plants-1168953-supplementary.pdf]

SUPPLEMENTARY MATERIAL

Article

Magnesium foliar supplementation increases grain yield of soybean and maize by improving photosynthetic carbon metabolism, and antioxidant metabolism

Vitor Alves Rodrigues [1], Carlos Alexandre Costa Crusciol [1,*] , João William Bossolani [1,*], Luiz Gustavo Moretti [1], José Roberto Portugal [1], Tamara Thaís Mundt [1], Sirlene Lopes de Oliveira [1], Ariani Garcia [1], Juliano Carlos Calonego [1] and Romulo Pisa Lollato [2]

[1] Department of Crop Science, College of Agricultural Sciences, São Paulo State University (UNESP), Botucatu, SP 18610-034, Brazil; souzamoretti@gmail.com (L.G.M.); jose.portugal@unesp.br (J.R.P.); tamarathaism@gmail.com (T.T.M.); sirlene.lopes@unesp.br (S.L.O.); ariani_garcia@hotmail.com (A.G.); juliano.calonego@unesp.br (J.C.C.).

[2] Throckmorton Plant Science Center, Department of Agronomy, Kansas State University (KSU), 1712, Claflin Road, Manhattan, KS 66506, United States; lollato@ksu.edu (R.P.L).

* Correspondence: carlos.crusciol@unesp.br (C.A.C.C.); bossolani.agro@gmail.com (J.W.B.).

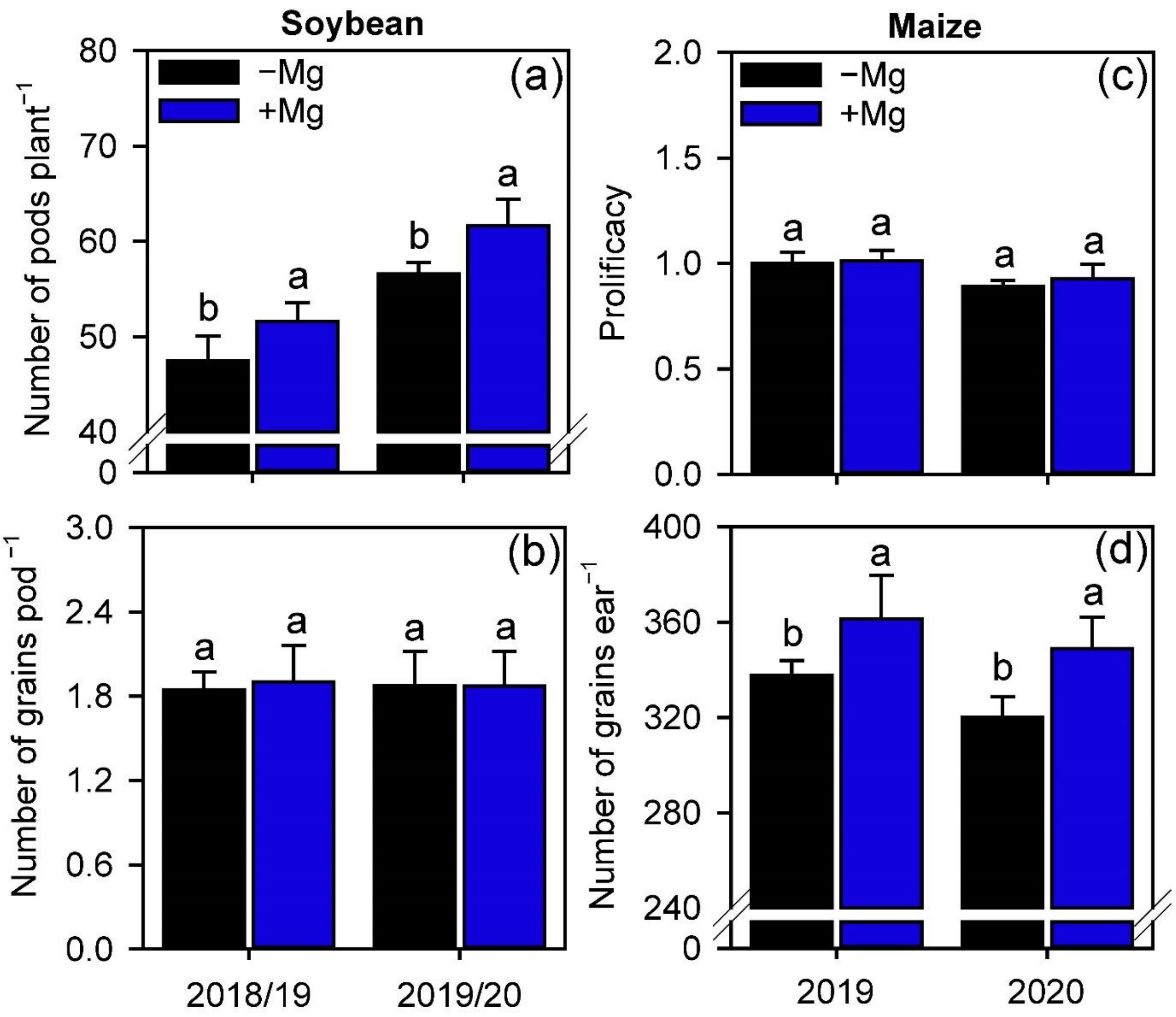

Figure S1. Number of pods per plant (**a**), and grains per pod (**b**) of soybean, and prolificacy (**c**), and number of grains per ear (**d**) of maize plants as affected by presence or absence of the foliar N application. Different lower-case letters indicate significant differences between treatments (presence or absence of Mg supplementation) by Fisher's protected LSD test at $p \leq 0.05$. Growing seasons was considered as random effects. Error bars express the standard error of the mean ($n = 4$).

Table S1. Physicochemical and biological attributes (0.0–0.2-m depth) before sowing.

Soil Properties	Unit	Value
**Physical**		
Clay	g kg^{-1}	602
Silt	g kg^{-1}	281
Sand	g kg^{-1}	117
Bulk density	g cm^{-3}	1.19
**Chemical**		
pH $_{(CaCl2)}$	–	5.10
Soil organic matter	g kg^{-1}	26.2
Phosphorus–available (P $_{resin}$)	mg kg^{-1}	59.0
Exchangeable		
Calcium ($Ca^{2+}{}_{resin}$)	$mmol_c$ kg^{-1}	25.0
Magnesium ($Mg^{2+}{}_{resin}$)	$mmol_c$ kg^{-1}	15.0
Potassium ($K^{+}{}_{resin}$)	$mmol_c$ kg^{-1}	3.90
Aluminum ($Al^{3+}{}_{KCl}$)	$mmol_c$ kg^{-1}	2.00
Potential acidity (H+Al)	$mmol_c$ kg^{-1}	42.0
S-Sulfate ($S–SO_4^{2-}{}_{Ca(H2PO4)2}$)	mg kg^{-1}	4.90
Boron (B $_{Hot\ water}$)	mg kg^{-1}	0.40
Copper (Cu $_{DTPA-TEA^a}$)	mg kg^{-1}	8.80
Iron (Fe $_{DTPA-TEA}$)	mg kg^{-1}	22.0
Manganese (Mn $_{DTPA-TEA}$)	mg kg^{-1}	26.2
Zinc (Zn $_{DTPA-TEA}$)	mg kg^{-1}	2.10
Base saturation (BS)	%	51.0
Cation exchange capacity (CEC $_{pH\ 7.0}$)	$mmol_c$ kg^{-1}	86.0
**Biological**		
Most probable number	CFU[b] g^{-1}	9.32×10^4

[a]DTPA-TEA, diethylenetriaminepentaacetic acid-triethanolamine;
